# Hydrogen gas inhalation alleviates oxidative stress in patients with post-cardiac arrest syndrome

**DOI:** 10.3164/jcbn.19-101

**Published:** 2020-04-03

**Authors:** Tomoyoshi Tamura, Masaru Suzuki, Kei Hayashida, Yosuke Kobayashi, Joe Yoshizawa, Takayuki Shibusawa, Motoaki Sano, Shingo Hori, Junichi Sasaki

**Affiliations:** 1 Department of Emergency and Critical Care Medicine, Keio University School of Medicine, Tokyo 160-8582, Japan; 2 The Center for Molecular Hydrogen Medicine, Keio University, Tokyo 108-8345, Japan; 3 Department of Cardiology, Keio University School of Medicine, Tokyo 160-8582, Japan

**Keywords:** molecular hydrogen gas, anti-oxidative effect, anti-inflammatory effect, target temperature management

## Abstract

Oxidative stress plays a key role in the pathophysiology of post-cardiac arrest syndrome. Molecular hydrogen reduces oxidative stress and exerts anti-inflammatory effects in an animal model of cardiac arrest. However, its effect on human post-cardiac arrest syndrome is unclear. We consecutively enrolled five comatose post-cardiac arrest patients (three males; mean age, 65 ± 15 years; four cardiogenic, one septic cardiac arrest) and evaluated temporal changes in oxidative stress markers and cytokines with inhaled hydrogen. All patients were treated with target temperature management. Hydrogen gas inhalation (2% hydrogen with titrated oxygen) was initiated upon admission for 18 h. Blood hydrogen concentrations, plasma and urine oxidative stress markers (derivatives of reactive oxygen metabolites, biological antioxidant potential, 8-hydroxy-2'-deoxyguanosine, *N*^ɛ^-hexanoyl-lysine, lipid hydroperoxide), and cytokines (interleukin-6 and tumor necrosis factor-α) were measured before and 3, 9, 18, and 24 h after hydrogen gas inhalation. Arterial hydrogen concentration was measurable and it was equilibrated with inhaled hydrogen. Oxidative stress was reduced and cytokine levels were unchanged in cardiogenic patients, whereas oxidative stress was unchanged and cytokine levels were diminished in the septic patient. The effect of inhaled hydrogen on oxidative stress and cytokines in comatose post-cardiac arrest patients remains indefinite because of methodological weaknesses.

## Introduction

Despite improvements in cardiopulmonary resuscitation and comprehensive post-arrest care, out-of-hospital cardiac arrest (OHCA) remains a major source of mortality in developed countries.^(1)^ Post-cardiac arrest syndrome (PCAS) comprises a complex combination of pathophysiological processes that are mainly characterized by brain and myocardial injury and a systemic ischemia/reperfusion (I/R) response.^(2)^ A dramatic increase in reactive oxygen species (ROS) generation occurs with the return of spontaneous circulation (ROSC), and oxidative stress elicited by ROS is recognized as having a central role in the development, progression, and pathophysiology of PCAS.^(2)^ Cardiac arrest (CA) and resuscitation results in an acute phase response that has been compared to sepsis, as PCAS is often referred to as a sepsis-like syndrome.^(3)^ I/R is associated with the activation of several interleukins (ILs), tumor necrosis factor (TNF)-α, and other cytokines.^(3–6)^ Of note, levels of inflammation are independently associated with increased mortality with OHCA.^(7)^

Target temperature management (TTM) is the only clinically-validated treatment option to improve neurological outcomes associated with PCAS.^(8,9)^ The neuroprotective mechanisms of TTM include the attenuation of oxidative stress and anti-inflammatory effects.^(10–13)^ However, recent studies reported that TTM does not exert beneficial effects on cytokine levels in PCAS.^(6,14–17)^

Molecular hydrogen (H_2_) is a mild antioxidant that selectively reduces highly active oxidants such as hydroxyl radicals and peroxynitrites.^(18)^ A growing body of evidence has indicated the pleiotropic effects of H_2_ including anti-inflammatory and anti-apoptotic effects, in addition to anti-oxidative effects after I/R.^(19–21)^ Inhaled H_2_ gas attenuates oxidative stress in the heart and brain, suppresses the activation of microglia and apoptosis, and improves neurological outcomes in a rodent cardiac arrest model.^(15,22)^ Further, the neuroprotective effects of perioperatively inhaled H_2_ gas were reported using a large animal porcine model of cardiopulmonary bypass.^(23)^ Recently, we also clinically translated the H_2_ gas inhalation method for humans with PCAS.^(24)^ Although reported in animal studies, the effect of H_2_ on oxidative stress and inflammatory cytokines in human PCAS has not been studied. Thus, the aim of the present study was to evaluate these effects using human PCAS subjects.

## Materials and Methods

### Study design

This was a sub-study of a single-center, open-label, single-arm, prospective intervention study, with the primary interest being the feasibility and safety of inhaled H_2_ gas for patients with PCAS (Clinical trial identifier: UMIN000012381).^(24)^ The study protocol was approved by the ethics committee of Keio University (Approval number: 20130075). Written informed consent was obtained from the patient’s family and from each patient who regained the necessary mental capacity.

### Selection of patients

We used the same subjects from a previous study.^(24)^ We consecutively enrolled five eligible patients between January 1, 2014 and January 19, 2015. The inclusion and exclusion criteria were reported previously.^(24)^ Briefly, adult patients between 20 and 80 years of age who experienced non-traumatic OHCA and sustained a coma, in which the individual could not follow verbal commands (Glasgow Coma Scale, ≤8) after successful resuscitation, were included. Patients with pre-existing severe neurological dysfunction, oxygen saturation less than 95% with 50% oxygen inhalation, systolic blood pressure less than 90 mmHg after adequate fluids and the use of catecholamines, and those who could not initiate H_2_ gas inhalation within 12 h of ROSC were excluded.

### Hydrogen gas inhalation

H_2_ gas inhalation (2% H_2_ with oxygen) was initiated using a ventilator system as previously described.^(24)^ H_2_ gas inhalation was initiated upon ICU admission and was continued for 18 h in conjunction with general post-arrest care (Supplemental Fig. 1*).

### General management of post-cardiac arrest patients

Multidisciplinary post-arrest care in accordance with the latest guidelines at the time was provided for each patient.^(1,25)^ All patients were managed with target temperature management between 34 and 36°C (see Supplementary materials* for detailed treatment protocols).

### Measurement of arterial hydrogen gas concentrations

Gas chromatography analysis was performed to determine the arterial H_2_ gas concentration as described previously.^(18)^ Briefly, 1 ml of arterial blood was drawn before and 3, 9, 18, and 24 h after the initiation of H_2_ gas inhalation. Drawn arterial blood was immediately injected into a closed aluminum bag containing 25 ml of air and stored at room temperature until analysis. After complete transfer of the H_2_ gas from the blood to the air in the closed bag, 1 ml of air containing H_2_ gas was drawn from the bag and H_2_ gas concentration was measured by gas chromatography analysis (Breath Gas Analyzer, Model TGA2000; TERAMECS Co. Ltd., Kyoto, Japan). Measurements were performed within 5 days of collection. The arterial H_2_ gas concentration was calculated based on comparisons with a calibration curve of standardized concentrations of H_2_ gas [standard H_2_ gas concentrations: 0.43, 0.78, 1.3, 2.5, 5, and 10% v/v (Taiyo Nippon Sanso Corporation, Tokyo, Japan)].

### Measurement of oxidative stress markers and inflammatory cytokines

Arterial blood was collected before and 3, 9, 18, and 24 h after the initiation of H_2_ gas inhalation. Immediately after collection, blood was centrifuged at 1,500 × *g* for 10 min at room temperature and collected plasma was snap frozen with liquid nitrogen and stored at –80°C until analysis. Urine was collected before and 24 h after the initiation of H_2_ gas inhalation through the urethral catheter and was frozen at –80°C. Plasma concentrations of oxidative stress markers and inflammatory cytokines were measured at the aforementioned five time points. Urine oxidative stress markers were measured before and 24 h after hydrogen gas inhalation. Oxidative stress markers and inflammatory cytokines were selected based on prior knowledge.^(3,6,11,15,16,22)^

Oxidative and anti-oxidative activities were assessed by measuring derivatives of reactive oxygen metabolites (dROMs) and biological antioxidant potential (BAP),^(26–29)^ respectively, using FREE Carpe Diem (Diacron International, Grosseto, Italy) as per the manufacturer’s instructions. Plasma and urine 8-hydroxy-2'-deoxyguanosine (8-OHdG), an index of oxidative DNA damage, as well as *N*^ɛ^-hexanoyl-lysine (HEL) and lipid hydroperoxide (LPO), an index of lipid peroxidation, were measured with respective ELISA kits at the Japan Institute for the Control of Aging (JaICA), Shizuoka, Japan. Urine 8-OHdG, HEL, and 8-isoprostane, also an index of lipid peroxidation, were measured with respective ELISA kits at JaICA, Shizuoka, Japan. Urine oxidative stress marker levels were corrected based on urine creatinine values. Plasma IL-6 and TNF-α were measured with a chemiluminescent enzyme immunoassay (Quanti Glo ELISA Human IL-6 Immunoassay and Quanti Glo ELISA Human TNF-α Chemiluminescent 2nd generation, respectively; both from R&D Systems, Minneapolis, MN). Plasma samples were obtained from all subjects (*n* = 5), and urine samples were obtained from four subjects.

### Statistical analysis

Variables were presented as the mean ± SD. A one-way repeated analysis of variance followed by a Tukey-Kramer multiple comparison test, paired *t* test, or Wilcoxon matched-pairs signed ranks-test was used when appropriate. Data are presented as the mean ± SD, unless otherwise indicated. A *p* value <0.05 was considered statistically significant. All statistical analyses were conducted using GraphPad Prism 8.0 (GraphPad Software Inc., La Jolla, CA).

## Results

### Patient characteristics

Patient characteristics were reported previously.^(24)^ Briefly, three patients (60%) were male, and the average age was 65 ± 15 years. One patient had a history of chronic kidney disease and was on maintenance hemodialysis, and other past medical histories are shown in Table 1. Four patients (80%) were diagnosed with cardiac etiology, and one patient experienced non-cardiogenic CA from hemodynamic deterioration due to sepsis with pneumonia. The estimated time from collapse to ROSC and ROSC to initiation of H_2_ gas inhalation was 16 ± 4.7 min and 4.9 ± 1.2 h, respectively. Four patients who experienced CA with a cardiac cause were treated with TTM at 34°C, and TTM at 36°C was selected for one patient with sepsis. The time interval from ROSC to reach target temperature was 6.4 ± 3.7 h. Respiratory conditions were stable up to 24 h after H_2_ gas inhalation (Table 2). The mean arterial pressure was well maintained in all patients during H_2_ gas inhalation and TTM with the continuous infusion of catecholamines (Table 3). Four patients with cardiac causes survived 90 days with a favorable neurological outcome (cerebral performance category of 1; Patients 2–5), whereas one patient with sepsis (Patient 1) died of worsened pneumonia 22 h after the discontinuation of H_2_ inhalation.^(24)^

### Blood hydrogen gas concentrations

**Fig. 1:**
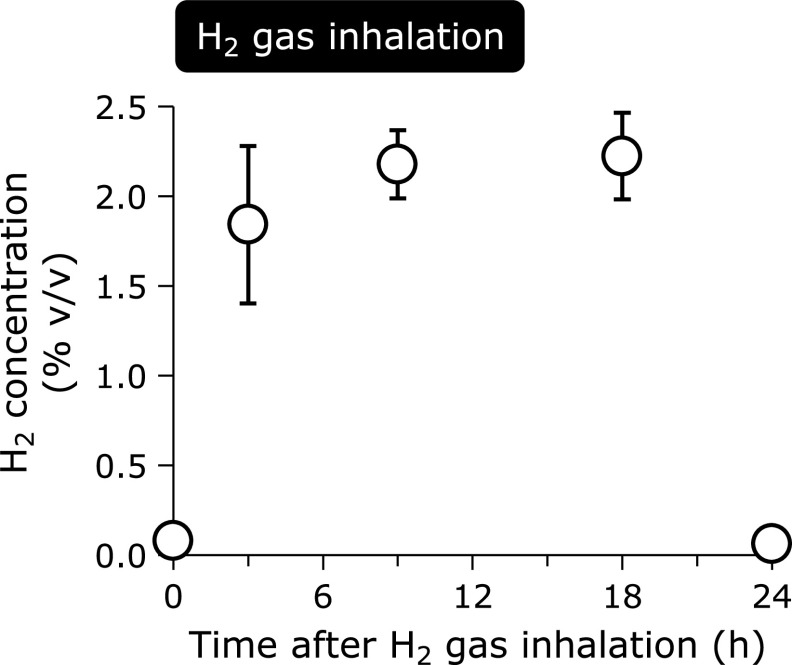
Arterial hydrogen concentrations in patients with post-cardiac arrest syndrome treated with H_2_ gas inhalation. Data are presented as the mean ± SD. H_2_ concentrations were measured by gas chromatography analysis based on comparisons with a calibration curve of standardized concentrations of H_2_ gas. Arterial H_2_ concentrations reached approximately 2% during inhalation. Arterial H_2_ concentrations decreased after discontinuing H_2_ gas inhalation. H_2_, molecular hydrogen; % v/v, volume percent.

Arterial H_2_ concentrations reached approximately 2% during H_2_ gas inhalation, but it was washed out after the discontinuation of inhalation at 24 h (Fig. 1). Measurements of arterial H_2_ at low concentrations were imprecise since it was calculated from the calibration curve.

### Temporal trends in oxidative stress markers

**Fig. 2:**
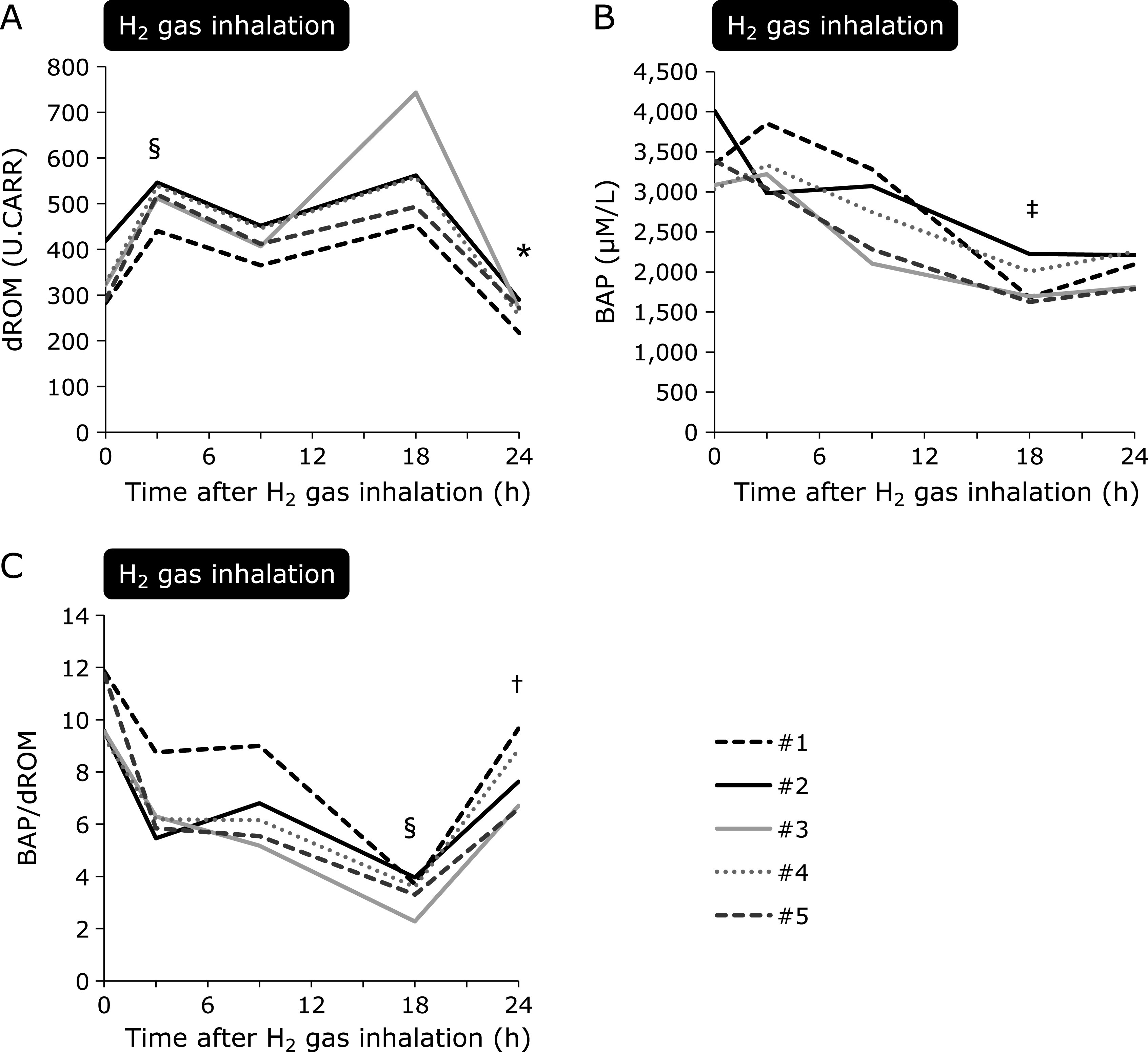
Changes in plasma dROMs and BAP with H_2_ gas inhalation. Each line indicates an individual patient. The black broken line indicates the patient with sepsis. (A) dROM values were 338.8 ± 55.8 U.CARR before H_2_ inhalation. After an increase to 511.4 ± 42.0 U.CARR at 3 h post-H_2_ gas inhalation (*p*<0.001, compared to 0 h), dROM levels were maintained during H_2_ gas inhalation but decreased to 271.4 ± 14.6 U.CARR 24 h after H_2_ gas inhalation (*p* = 0.01, compared with 18 h). (B) BAP was 3,379.6 ± 450.6 µM/L before H_2_ gas inhalation, and it decreased during H_2_ gas inhalation (*p* = 0.001, compared to 0 h). In contrast to that observed for dROMs, a slight increase in BAP levels was observed after the discontinuation of H_2_ gas inhalation (2,014.9 ± 251.4 µM/L at 24 h; *p* = 0.23, compared to 18 h). (C) The BAP to dROM ratio was reduced during H_2_ inhalation (*p*<0.001) and increased after completing H_2_ gas inhalation (*p* = 0.004, 18 h vs 24 h). All patients were still being treated with target temperature management (TTM) at 24 h. *p = 0.01, compared to 18 h; ^†^*p* = 0.004, compared to 18 h; ^‡^*p* = 0.001, compared to 0 h; ^§^*p*<0.001, compared to 0 h. dROMs, derivatives of reactive oxygen metabolites; BAP, biological antioxidant potential; H_2_, molecular hydrogen.

dROM was significantly increased 3 h post-H_2_ gas inhalation and was maintained during H_2_ gas inhalation but decreased at 24 h after H_2_ gas inhalation (Fig. 2A). BAP significantly decreased during H_2_ gas inhalation. In contrast to that observed for dROM, a slight increase in BAP levels was observed after the discontinuation of H_2_ gas inhalation (Fig. 2B). The BAP over dROM ratio, which indicates a relative tolerance to oxidative stress, was decreased approximately linearly between 3 and 18 h of H_2_ gas inhalation and sharply increased after completing H_2_ gas inhalation (Fig. 2C). Of note, all patients were treated with TTM during H_2_ gas inhalation and body temperature was maintained at the target temperature (maintenance phase) between 6.4 and 24 h of the observation period (Supplemental Fig. 1*).

**Fig. 3:**
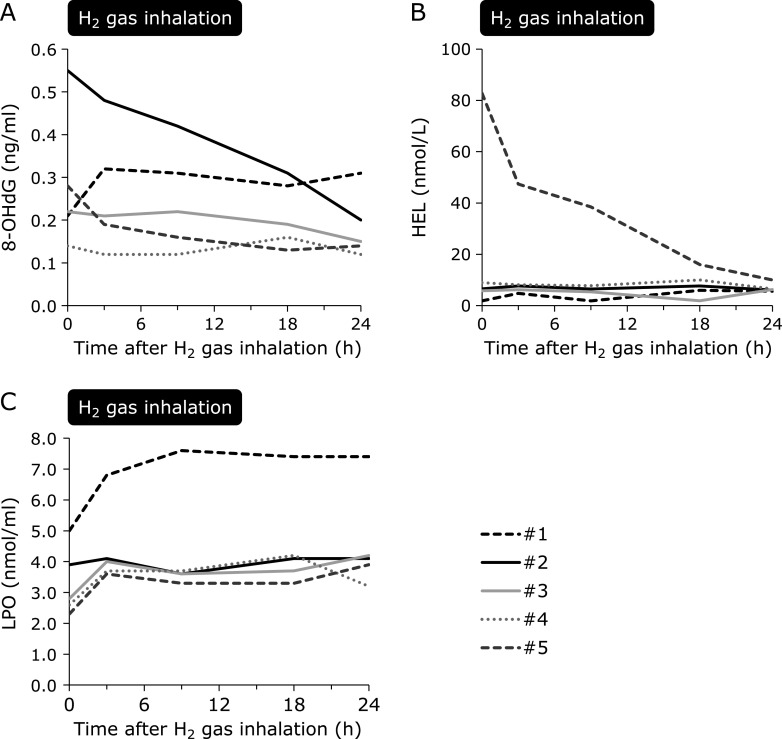
Temporal changes in plasma oxidative stress markers in patients with post-cardiac arrest syndrome treated with H_2_ gas inhalation. Each line indicates an individual patient. (A) 8-OHdG, a marker of DNA damage, was decreased, except for in one patient with sepsis, from 0.30 ± 0.18 ng/ml to 0.15 ± 0.03 ng/ml before H_2_ gas inhalation and at 24 h, respectively (*p* = 0.31). (B) HEL, an early marker of lipid peroxidation, decreased dramatically in one patient, but remained the same in other patients (*p* = 0.39). (C) LPO, a marker of lipid peroxidation, slightly increased within 3 h after the initiation of H_2_ gas inhalation (*p* = 0.05, 0 h vs 3 h), but was then maintained for 24 h (*p* = 0.96, 3 h vs 24 h). LPO was markedly high in the patient with sepsis. 8-OHdG, 8-hydroxy-2'-deoxyguanosine; H_2_, molecular hydrogen; HEL, *N*^ɛ^-hexanoyl-lysine; LPO, lipid hydroperoxide.

**Fig. 4:**
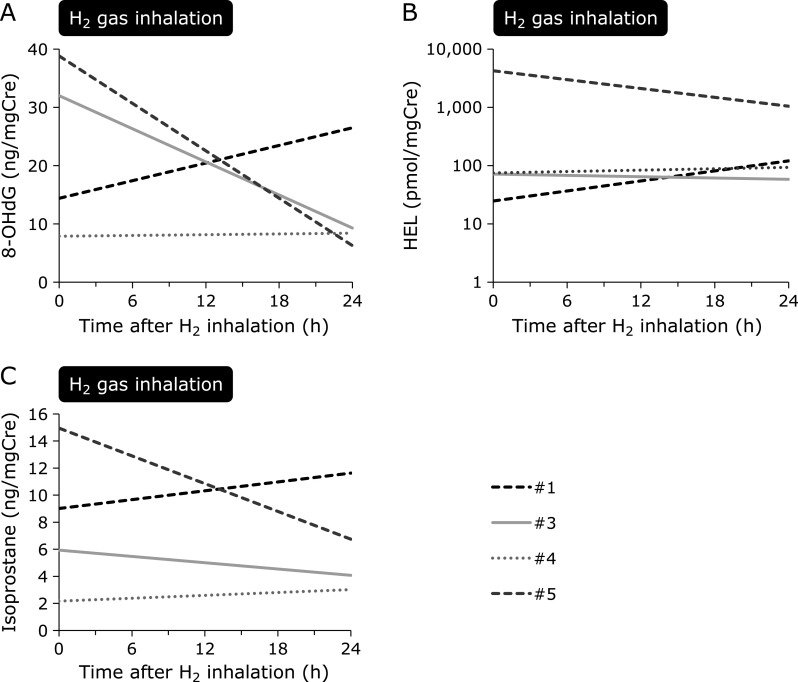
Temporal changes in urine oxidative stress markers in patients with post-cardiac arrest syndrome treated with H_2_ gas inhalation. Each line indicates an individual patient. Urine was collected from the urinary catheter before and 24 h after H_2_ gas inhalation. Urine could not be assessed in one patient who was anuric and on maintenance hemodialysis. (A) Urine 8-OHdG, a marker of DNA damage, increased in the patient with sepsis, but decreased in the other patients (26.2 ± 16.2, 8.0 ± 1.5 ng/mgCre; 0 h and 24 h, respectively, *p* = 0.20). (B) Urine HEL, an early marker of lipid peroxidation, also increased in the patient with sepsis, but decreased in the other patients (1,463.7 ± 2,407.9, 401.0 ± 563.0 pmol/mgCre; 0 h and 24 h, respectively, *p* = 0.59). (C) Urine isoprostane, a marker of lipid peroxidation, was elevated in the patient with sustained sepsis, whereas it slightly decreased in the other patients (7.7 ± 6.6, 4.6 ± 1.9 ng/mgCre; 0 h and 24 h, respectively, *p* = 0.37). 8-OHdG, 8-hydroxy-2'-deoxyguanosine; Cre, creatinine; H_2_, molecular hydrogen; HEL, *N*^ɛ^-hexanoyl-lysine.

Except for those in one patient with sepsis, despite a difference in the degree, 8-OHdG levels decreased after H_2_ gas inhalation at 24 h (Fig. 3A). HEL decreased dramatically in one patient but was maintained at the same levels in other patients (Fig. 3B). LPO slightly increased at 3 h after the initiation of H_2_ gas inhalation but was then maintained over 24 h. Compared to levels of other measured oxidative stress markers, LPO was markedly high in the patient with sepsis (Fig. 3C). No apparent change in 8-OHdG, HEL, and LPO, as compared to that for dROM and BAP, was observed after discontinuing H_2_ gas inhalation. Urine samples were examined from four patients, excluding one anuric patient with chronic kidney disease who was on maintenance hemodialysis. Urine 8-OHdG, HEL, and isoprostane levels increased 24 h after H_2_ gas inhalation in one patient with sepsis (Fig. 4), whereas urine oxidative stress markers were reduced or maintained at the same level 24 h after H_2_ gas inhalation in the other three cardiogenic post-CA patients (Fig. 4).

### Temporal trends in inflammatory cytokines

**Fig. 5:**
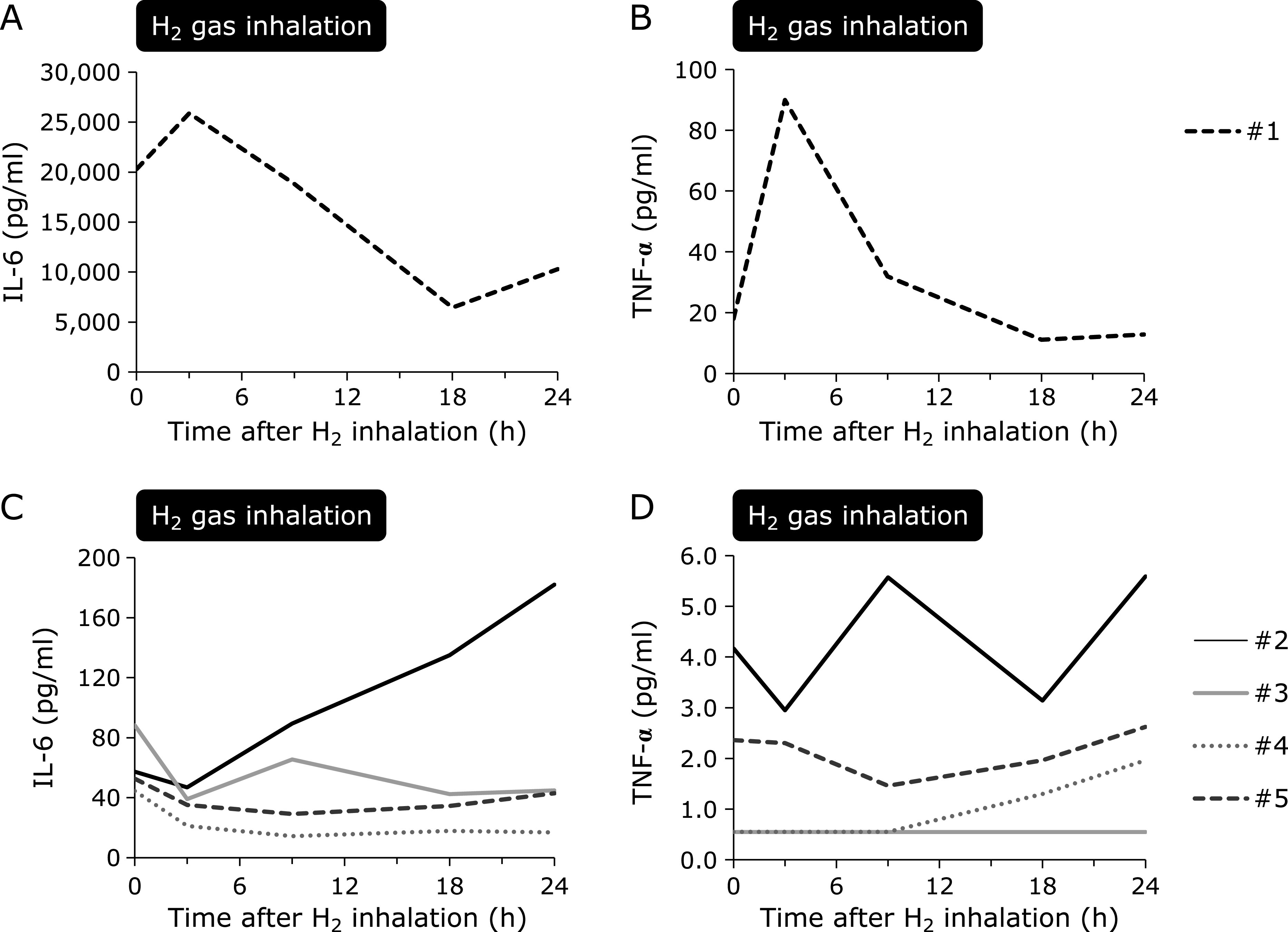
Temporal changes in inflammatory cytokines in patients with post-cardiac arrest syndrome treated with H_2_ gas inhalation. Each line indicates an individual patient. (A) IL-6, and (B) TNF-α levels were markedly high in one patient with sepsis. Although both IL-6 and TNF-α increased within 3 h after H_2_ gas inhalation, they were subsequently decreased drastically. After completing H_2_ inhalation, IL-6 and TNF-α levels increased again. (C) IL-6 and (D) TNF-α levels were low among the other four patients. Both IL-6 and TNF-α increased in one patient with a history of end-stage kidney disease treated with maintenance hemodialysis. IL-6 and TNF-α levels were maintained at similar levels over 24 h in the other three patients (*p* = 0.13, *p* = 0.28, respectively). H_2_, molecular hydrogen; IL-6, interleukin-6; TNF-α, tumor necrosis factor-α.

Plasma IL-6 and TNF-α levels were markedly high in the patient with sepsis, as compared to those in the other cardiogenic post-CA patients (Fig. 5). In the patient with sepsis, IL-6 and TNF-α were significantly attenuated during H_2_ gas inhalation and increased after its discontinuation (Fig. 5A and B). Levels of IL-6 and TNF-α were low among patients with cardiogenic post-CA and were comparable during H_2_ gas inhalation. In contrast, IL-6 and TNF-α were higher in one cardiogenic post-CA patient undergoing maintenance hemodialysis and increased during the observational period in this individual (Fig. 5C and D).

## Discussion

In the present study, the temporal changes in oxidative stress and inflammatory cytokines upon H_2_ gas inhalation were sequentially measured in patients with PCAS. The results revealed that oxidative stress markers were reduced in cardiogenic post-CA patients but were slightly elevated in the patient with sepsis with H_2_ gas inhalation. Inflammatory cytokine levels remained unchanged in cardiogenic post-CA patients, whereas a dramatic reduction was observed in one patient with sepsis. To the best of our knowledge, this is the first study to report changes in oxidative stress markers and inflammatory cytokines in PCAS patients undergoing H_2_ gas inhalation.

Although animal studies suggested the association between cardiac arrest and oxidative stress,^(30,31)^ scarce data existed with regard to changes in oxidative stress in human PCAS. It was previously reported that TTM at 33°C reduces both dROM and BAP in PCAS patients.^(11)^ This result is consistent with our findings; specifically, dROM and BAP were both reduced at 24 h after the initiation of H_2_ gas inhalation, where patients finished H_2_ gas inhalation at 18 h but were still treated at the target temperature at this point. However, opposite to our expectations, a slight increase in dROM, a decrease in BAP, and a decrease in the BAP to dROM ratio was also observed. It is noteworthy that all patients showed the same temporal change regardless of differences in target temperatures used and cardiac arrest etiologies. Since the patient with sepsis was treated with normothermia, these changes in dROM and BAP might not be solely due to hypothermia. Moreover, the observed sharp increase in the BAP to dROM ratio after finishing H_2_ gas inhalation, even upon treatment with the target temperature, might be attributed to the cessation of H_2_ gas inhalation. However, the reason for these findings needs to be elucidated in future studies.

It was previously reported that small differences can be observed in thiobarbituric acid reactive species among favorable and poor neurological outcome groups early after ROSC, but that other oxidative stress markers and antioxidant enzyme levels are not significantly different.^(32)^ In another study, increased oxidative stress, indicated by increased plasma coenzyme Q10 and free fatty acids, was reported in post-cardiac arrest patients.^(33)^ Time-course changes in several lipids and antioxidants were also assessed, but with a lack of comparisons using definitive endpoints, these results have limited clinical value. The optimal mode and timing for measuring oxidative stress markers in human PCAS is unclear. Therefore, we measured a panel of oxidative stress markers in an exploratory attempt. An association between elevated 8-OHdG levels and cardiovascular disease is known to occur in patients,^(34,35)^ and H_2_ inhalation was previously reported to attenuate 8-OHdG production in the heart and brain based on animal models of I/R injury.^(15,19)^ With H_2_ gas inhalation, our results showed a decrease in both plasma and urine 8-OHdG levels during the first 24 h after admission.

Lipid peroxidation is another important oxidative stress that occurs after CA.^(36,37)^ Recently, HEL was identified as a novel lipid hydroperoxide-modified lysine residue that is produced in the earlier phase of lipid peroxidation, as compared to traditionally-studied malondi-aldehyde and 4-hydroxy-2-nonenal, which are generated at the later stage of this process.^(38)^ Regarding this marker, our results revealed a drastic decrease in one patient, with no significant increases observed in the other patients. LPO, another marker of lipid peroxidation, was also maintained at a steady level. Of note, isoprostane, a product of the free radical-catalyzed peroxidation of arachidonic acid,^(21,39)^ decreased with H_2_ gas inhalation; this was thought to be because arachidonic acid is a main component of phospholipids and H_2_ has been reported to suppress the free radical chain reaction-dependent peroxidation of phospholipids.^(40)^ However, all patients underwent TTM, which is also known to alleviate lipid peroxidation,^(36)^ and since there was no obvious change at 18 h, the net effect of inhaled H_2_ in addition to TTM requires further study.

The association between elevated cytokine levels and poor outcomes was reported previously for PCAS.^(6,7)^ Interestingly, TTM failed to lower cytokine levels, and IL-6 levels were even reported to be elevated during this treatment.^(6,14)^ H_2_ gas inhalation was reported to lower IL-6 levels in an animal cardiac arrest model.^(15)^ Overall cytokine levels were low and remained unchanged in patients with cardiac etiologies, suggesting that inhaled hydrogen might have attenuated the elevation in cytokines at an early phase with TTM. IL-6 was slightly elevated in one patient who was undergoing maintenance hemodialysis. However, pro-inflammatory cytokines are known to be higher in patients undergoing hemodialysis.^(41–43)^ The fact that hemodialysis was not performed during the 24 h after admission might have contributed to the increase in pro-inflammatory cytokines in this patient. Further, one patient who experienced CA from sepsis showed incommensurable high cytokine levels compared to those in patients with cardiac etiologies. However, cytokine levels were drastically decreased upon H_2_ gas inhalation in this patient and a rebound was observed after the discontinuation of this treatment. These observations suggest the anti-inflammatory effect of H_2_.

We acknowledge several limitations to this study. Since this was a sub-study comprising a first-in-human PCAS study to evaluate the feasibility and safety of H_2_ inhalation, it lacks a control group who did not inhale H_2_. Moreover, oxidative stress markers and inflammatory cytokines were observed only for the first 24 h, while patients were subjected to TTM, and data after rewarming are lacking. Moreover, outcomes were assessed at 90 days in this study, and therefore, future studies are needed to evaluate the longer-term effect of inhaled H_2_ on cognitive function or quality of life. Due to the small sample size and overall high percentage of favorable outcomes, levels of cytokines were low compared to those in previous reports.^(7,44)^ To further enhance our understanding of this treatment modality, we are currently conducting a large, multicenter, double-blind, randomized control trial in Japan,^(45)^ and the results of this study are expected to reveal the anti-oxidative and anti-inflammatory effect of inhaled H_2_.

In the present study, temporal changes in blood H_2_ concentration, oxidative stress, and inflammatory cytokine levels with H_2_ gas inhalation were assessed in human PCAS. This was the first human study to evaluate oxidative stress and cytokine levels in PCAS patients who inhaled H_2_. Further clinical studies are needed to evaluate the effect of H_2_ gas on oxidative stress and inflammatory cytokines in these individuals.

## Author Contributions

Study concept and design, KH, MSuzuki, SH; acquisition of data, TT, KH, JY, TS; analysis and interpretation of data, TT, KH, MSuzuki; drafting of the manuscript, TT; critical revision of the manuscript for important intellectual content, KH, JY, TS, MSuzuki, MSano, JS; statistical analysis, TT; obtained funding, SH.

## Figures and Tables

**Table 1:** Past medical history

Patient	Active	Inactive
1	None	
2	AF, CHF, ESRD	Rectal cancer
3	None	
4	GERD, HOCM, hypertension	Psoriatic arthritis
5	Dyslipidemia, HOCM	ASD, IE

AF, atrial fibrillation; ASD, atrial septum defect; CHF, chronic heart failure; ESRD, end stage renal disease; GERD, gastroesophageal reflux disease; HOCM, hypertrophic obstructive cardiomyopathy; IE, infectious endocarditis.

**Table 2:** Arterial gas analysis in patients with post-cardiac arrest syndrome

	Admission	3 h	9 h	18 h	24 h
FiO_2_	0.48 ± 0.04	0.48 ± 0.04	0.48 ± 0.04	0.48 ± 0.04	0.52 ± 0.2
PEEP (cm H_2_O)	6.0 ± 2.2	6.0 ± 2.2	6.0 ± 2.2	6.0 ± 2.2	7.0 ± 2.7
pH	7.366 ± 0.08	7.344 ± 0.1	7.409 ± 0.06	7.364 ± 0.05	7.391 ± 0.09
PCO_2_ (mmHg)	43.3 ± 7.9	42.4 ± 4.6	39.0 ± 6.0	42.7 ± 4.5	41.7 ± 9.5
PO_2_ (mmHg)	136 ± 14	147 ± 53	135 ± 31	150 ± 64	126 ± 26
HCO_3_^−^ (mmol/L)	23.9 ± 1.0	22.2 ± 3.2	24.0 ± 1.4	23.7 ± 1.1	24.3 ± 0.6
B.E. (mmol/L)	–0.8 ± 1.8	–2.4 ± 3.6	–0.1 ± 1.6	–0.9 ± 1.5	–0.6 ± 1.7
Lactate (mmol/L)	1.38 ± 0.4	5.08 ± 6.6	2.72 ± 2.0	2.5 ± 2.4	1.45 ± 0.5

Hours indicate time after the initiation of hydrogen gas inhalation. B.E., base excess; FiO_2_, fraction of oxygen; HCO_3_^−^, bicarbonate ion; PCO_2_, pressure of carbon dioxide; PEEP, positive end expiratory pressure; PO_2_, pressure of oxygen.

**Table 3:** Mean arterial pressure and catecholamine dosage in patients with post-cardiac arrest syndrome

	Admission	3 h	9 h	18 h	24 h
MAP (mmHg)	88.0 ± 12.3	85.9 ± 22.8	91.1 ± 20.0	94.3 ± 11.4	88.0 ± 24.6
Norepinephrine (µg/kg/min)	0.06 ± 0.1	0.1 ± 0.1	0.1 ± 0.1	0.05 ± 0.1	0.1 ± 0.1
Dopamine (µg/kg/min)	0	0	0	0.6 ± 1.3	1.6 ± 3.6
Dobutamine (µg/kg/min)	0.6 ± 1.3	0.6 ± 1.3	0.6 ± 1.3	0.5 ± 1.1	0

Hours indicate time after the initiation of hydrogen gas inhalation. MAP, mean arterial pressure.

## Abbreviations

BAP: biological antioxidant potential
dROMs: derivatives of reactive oxygen metabolites
H_2_: molecular hydrogen
HEL: *N*^ɛ^-hexanoyl-lysine
IL: interleukins
I/R: ischemia/reperfusion
LPO: lipid hydroperoxide
OHCA: out-of-hospital cardiac arrest
8-OHdG: 8-hydroxy-2'-deoxyguanosine
PCAS: post-cardiac arrest syndrome
ROS: reactive oxygen species
ROSC: return of spontaneous circulation
TNF-α: tumor necrosis factor-α
TTM: target temperature management
