# Supplementary material for: Hydrogen gas inhalation alleviates oxidative stress in patients with post-cardiac arrest syndrome
Source: J Clin Biochem Nutr. 2020 Apr 3;67(2):214–21. doi: 10.3164/jcbn.19-101 (PMC7533855; doi:10.3164/jcbn.19-101)
Supplement: Supplementary Methods [file jcbn19-101sm01.pdf]

Supplementary Methods

Detailed general management of post-cardiac arrest patients.

Patients were intubated and mechanically ventilated. Pressure-controlled ventilation was additionally chosen during hydrogen gas inhalation. Continuous intravenous infusions of midazolam and/or propofol and fentanyl were used for sedation and analgesia, respectively. A neuromuscular blocking agent, rocuronium, was used during hydrogen gas inhalation for all patients to allow mandatory volume-controlled ventilation. Continuous intravenous infusion of the neuromuscular blocking agent was terminated after the completion of hydrogen gas inhalation for patients treated with target temperature management (TTM) at 36°C and after rewarming to 36°C for patients who underwent TTM at 34°C. Norepinephrine, dopamine, and/or dobutamine were given when needed to maintain mean arterial pressure >65 mmHg.

All patients were managed with TTM between 34 and 36°C. TTM at 34°C was chosen unless otherwise contraindicated (i.e., for overt bleeding diathesis, uncontrollable electronical or hemodynamical instability, and infection). The administration of cold crystalloids and a neuromuscular blocking agent, in addition to a surface cooling device (Arctic Sun 2000®, Medivance Inc., Louisville, CO), were used in combination to reach the target temperature as soon as possible. The target temperature was maintained for 24 h and then patients were rewarmed to 36°C over the next 48 h. For patients treated with TTM at 36°C, the temperature was maintained at 36°C for 72 h.
