# Supplementary material for: Hydrogen gas inhalation alleviates oxidative stress in patients with post-cardiac arrest syndrome
Source: J Clin Biochem Nutr. 2020 Apr 3;67(2):214–21. doi: 10.3164/jcbn.19-101 (PMC7533855; doi:10.3164/jcbn.19-101)
Supplement: Supplemental Figure 1 [file jcbn19-101sf01.pdf]

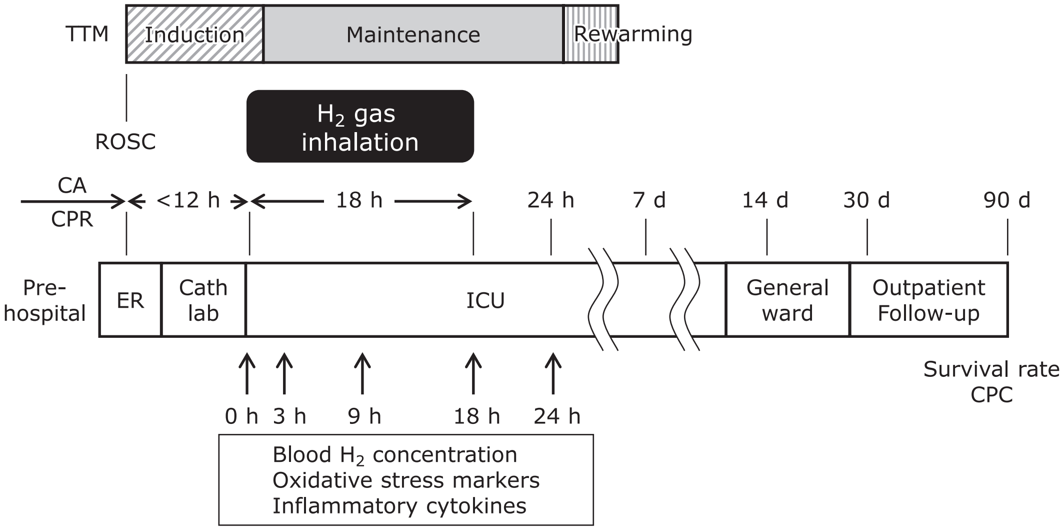

Supplemental Fig. 1. Overview of the study protocol. H_2 gas inhalation was initiated upon admission to the ICU and was continued along with other post-arrest care. All patients were treated with TTM. Arterial H_2 gas concentration, oxidative stress markers, and inflammatory cytokine levels were measured before and 3, 9, 18, and 24 h after the initiation of H_2 gas inhalation. Urine oxidative stress markers were also measured before and 24 h after the initiation of hydrogen gas inhalation. CA, cardiac arrest; cath lab, catheterization laboratory; CPC, cerebral performance category; CPR, cardiopulmonary resuscitation; ER, emergency room; H_2, molecular hydrogen; ICU, intensive care unit; ROSC, return of spontaneous circulation; TTM, target temperature management.
